# YAP/TAZ Signaling as a Molecular Link between Fibrosis and Cancer

**DOI:** 10.3390/ijms19113674

**Published:** 2018-11-20

**Authors:** Satoshi Noguchi, Akira Saito, Takahide Nagase

**Affiliations:** 1 Department of Respiratory Medicine, Graduate School of Medicine, The University of Tokyo, 7-3-1 Hongo, Bunkyo-ku, Tokyo 113-0033, Japan; asaitou-tky@umin.ac.jp (A.S.); takahide-tky@umin.ac.jp (T.N.); 2 Division for Health Service Promotion, The University of Tokyo, 7-3-1 Hongo, Bunkyo-ku, Tokyo 113-0033, Japan

**Keywords:** YAP, TAZ, Hippo pathway, fibrosis, cancer, mechanotransduction, TGF-β, Wnt

## Abstract

Tissue fibrosis is a pathological condition that is associated with impaired epithelial repair and excessive deposition of extracellular matrix (ECM). Fibrotic lesions increase the risk of cancer in various tissues, but the mechanism linking fibrosis and cancer is unclear. Yes-associated protein (YAP) and the transcriptional coactivator with PDZ-binding motif (TAZ) are core components of the Hippo pathway, which have multiple biological functions in the development, homeostasis, and regeneration of tissues and organs. YAP/TAZ act as sensors of the structural and mechanical features of the cell microenvironment. Recent studies have shown aberrant YAP/TAZ activation in both fibrosis and cancer in animal models and human tissues. In fibroblasts, ECM stiffness mechanoactivates YAP/TAZ, which promote the production of profibrotic mediators and ECM proteins. This results in tissue stiffness, thus establishing a feed-forward loop of fibroblast activation and tissue fibrosis. In contrast, in epithelial cells, YAP/TAZ are activated by the disruption of cell polarity and increased ECM stiffness in fibrotic tissues, which promotes the proliferation and survival of epithelial cells. YAP/TAZ are also involved in the epithelial–mesenchymal transition (EMT), which contributes to tumor progression and cancer stemness. Importantly, the crosstalk with transforming growth factor (TGF)-β signaling and Wnt signaling is essential for the profibrotic and tumorigenic roles of YAP/TAZ. In this article, we review the latest advances in the pathobiological roles of YAP/TAZ signaling and their function as a molecular link between fibrosis and cancer.

## 1. Introduction

Fibrosis is a pathological process that is characterized by mesenchymal cell infiltration and proliferation in the interstitial space. The fibrogenic response consists of the following phases: (1) initiation of the response driven by primary injury to the organ, (2) activation of effector cells such as fibroblasts induced by inflammatory mediators, and (3) elaboration of the extracellular matrix (ECM) [1]. Under normal conditions, the repair process is completed by provisional ECM degradation and the removal of excessive mesenchymal cells by apoptosis and phagocytosis, which leaves minimal damage and restores the normal architecture of the tissue. However, dysregulated processes can result in dynamic deposition and an insufficient resorption of ECM, which promotes progression to fibrosis and, ultimately, to end-organ failure. The transforming growth factor (TGF)-β and Wnt signaling pathways play pivotal roles in fibroblast activation and ECM deposition, and these pathways are reported to be activated in human fibrotic tissue as well as experimental models of fibrosis [2,3].

Fibrotic lesions increase the risk of cancer in various tissues, including the lung [4] and liver [5]. However, the mechanism linking fibrosis and cancer is unclear. The formation of fibrotic ECM disrupts epithelial cell polarity, and the abundance of growth factors in the profibrotic milieu stimulates the proliferation of epithelial cells, creating conditions that are favorable for cancer initiation and progression. Fibrosis and cancer have several dysregulated intercellular communication and intracellular signaling pathways in common. Indeed, cancer stroma displays fibrotic reactions to various degrees, which is termed desmoplasia, as does organ fibrosis. Of note, the TGF-β and Wnt signaling pathways are activated in cancer tissues, which are likely to contribute to cancer progression.

The Hippo pathway is an evolutionarily conserved signaling pathway that has multiple biological functions in development, homeostasis, and regeneration of tissues and organs. Yes-associated protein (YAP) and its paralog, the transcriptional coactivator with PDZ-binding motif (TAZ), which is also known as the WW-domain containing transcription regulator-1 (WWTR1), are core components of the Hippo pathway [6,7,8,9]. While YAP and TAZ have a lot of similarities in their structures, regulations, and functions, they have distinct and non-overlapping roles. YAP/TAZ signaling is involved in both fibrosis and cancer. Accumulating evidence indicates that YAP/TAZ act as sensors of mechanical forces and modulate the fibrotic response as well as the behavior of cancer cells. Moreover, YAP/TAZ function in a cooperative manner with other established signaling pathways. In particular, the crosstalk with the TGF-β and Wnt signaling pathways is closely associated with both fibrosis and cancer [10,11]. Here, we review the recent progress in the pathobiological role of YAP/TAZ signaling and its function as a molecular link between fibrosis and cancer.

## 2. Overview of YAP/TAZ Signaling

The core components of the Hippo pathway were initially identified by genetic screens to identify tumor suppressors in *Drosophila*, and ‘Hippo’ originated from the morphological phenotype of a *Drosophila* mutant [12]. Subsequent cellular and genetic studies have demonstrated that the core components of the Hippo pathway are highly conserved from *Drosophila* to mammals. The mammalian Hippo pathway includes a kinase cascade of mammalian sterile 20-like kinase 1/2 (MST1/2) and large tumor suppressor kinase 1/2 (LATS1/2). MST1/2 in complex with the regulatory protein SAV1 phosphorylate hydrophobic motifs of LATS1/2, which form a complex with the regulatory protein, MOB1 [13]. Phosphorylated and activated LATS1/2 then phosphorylate serine residues of YAP/TAZ. Upon phosphorylation by LATS1/2, YAP/TAZ interact with 14-3-3, which sequesters YAP/TAZ from nuclear translocation, leading to ubiquitination-mediated proteasomal and autolysosomal degradation [14]. The phosphorylation of YAP/TAZ results in the loss of their transcriptional coactivator function. In contrast, unphosphorylated YAP/TAZ localize to the nucleus, and act mainly through TEAD family transcription factors (TEADs) to stimulate the expression of genes—including CTGF, AXL, BIRC5, and AREG—involved in cell proliferation and the suppression of apoptosis [15]. In addition to TEADs, YAP/TAZ also interact with other transcription factors—such as Smad, Runx2, p73, and TBX5—to mediate cellular context-dependent transcriptional regulation [16]. As a negative regulator of the YAP–TEAD transcriptional complex, VGLL4 directly competes with YAP for binding to TEADs [17].

A variety of upstream signals activate or inhibit YAP/TAZ signaling. Apical-basal polarity regulates YAP/TAZ subcellular localization and activity through interactions with cell-polarity proteins (Scribble and Crumbs) or cell-junction molecules (angiomotin and α-catenin) [18]. Extracellular hormones modulate LATS1/2 kinase activity via G protein-coupled receptor (GPCR) signaling [19]. Serum-borne lysophosphatidic acid (LPA) and sphingosine-1-phosphophate (S1P) act through G12/13-coupled receptors to inhibit LATS1/2, thereby activating YAP/TAZ. Furthermore, recent evidence has shown that a variety of stress signals—such as energy stress, endoplasmic reticulum stress, oxidative stress, and hypoxia—regulate the activity of YAP/TAZ [20,21,22,23].

## 3. Mechanotransduction and YAP/TAZ Activity

In addition to the above-mentioned upstream signals, extracellular mechanical cues including ECM stiffness, cell attachment or detachment, and cellular tension are potent regulators of YAP/TAZ. Dupont et al. first reported the association of YAP/TAZ activity with ECM stiffness and cell spreading [24]. In cells stretched by a stiff ECM, YAP/TAZ localize predominantly to the nucleus, and their transcriptional activity is elevated. On the other hand, their localization is predominantly cytoplasmic on a soft ECM. This regulation is dependent on Rho GTPase and the tension of the actomyosin cytoskeleton. Notably, this process is independent of LATS1/2, because the depletion of LATS1/2 had a marginal effect on the regulation of YAP/TAZ activity by mechanical cues. The LATS1/2-dependent regulation of YAP/TAZ activity by stress fiber (F-actin) formation has been reported [25,26]. This finding was confirmed by the observation that the F-actin-capping/severing proteins cofilin, CapZ, and gelsolin restrict the nuclear localization of YAP [27]. Zhao et al. showed that cell detachment from ECM activates LATS1/2 by promoting cytoskeleton reorganization, which leads to YAP inactivation and apoptosis, which is a process termed anoikis [28].

The mechanisms by which cytoskeletal tension regulates YAP/TAZ are unclear, although the nucleus may play a mechanotransductive role in the regulation of YAP [29]. The focal adhesions and stress fibers that are generated on stiff substrates transduce mechanical forces to the nucleus, leading to nuclear flattening. This increases YAP nuclear import by reducing mechanical restriction in nuclear pores. In contrast, on soft substrates, mechanical forces fail to reach the nucleus, and nucleocytoplasmic shuttling of YAP through nuclear pores is balanced.

Interactions between cells and ECM are largely mediated by the proteins of the integrin family. Focal adhesions composed of integrins, focal adhesion kinase (FAK), and Src play an important role as a sensor of ECM rigidity and in the intracellular transduction of extracellular signals. FAK stimulates Rho-associated protein kinase (ROCK)-dependent actin remodeling and the formation of stress fibers. Integrin-linked kinase (ILK) inactivates NF2, which is an upstream negative regulator of YAP/TAZ signaling, by inhibiting MYPT1 via direct phosphorylation [30]. The integrin-dependent FAK–Src signaling pathway also positively regulates the activity of YAP/TAZ. Integrin-dependent adhesion leads to the activation of p21-activated kinase 1 (PAK1), which is a downstream effector of the small GTPase RAC1. PAK1 directly phosphorylate NF2, which reduces its interactions with YAP, resulting in YAP dephosphorylation and nuclear translocation [31]. Adhesion to fibronectin also enhances YAP nuclear translocation via the FAK–Src–PI3K–PDK1 pathway [32]. However, despite these significant roles of the integrin signaling, cells spreading over polylysine-coated cover glasses, where cells do not form focal adhesions, show nuclear translocation of YAP, suggesting that focal adhesion formation is not always required for YAP mechanoactivation [28]. Interestingly, YAP/TAZ directly regulate a number of proteins involved in focal adhesion and control cytoskeleton stability [33].

## 4. YAP/TAZ Signaling in Fibrosis

Fibrosis is a sequela of several chronic inflammatory diseases, and progressive fibrosis typically has a devastating clinical course without good therapeutic options. Injury to epithelial cells results in the release of a variety of cytokines, chemokines, and growth factors. These mediators induce fibroblast accumulation and convert fibroblasts into myofibroblasts positive for α-smooth muscle actin (α-SMA). The epithelial repair is facilitated through mutual communication between the regenerating epithelium and activated fibroblasts. In the pathogenesis of tissue fibrosis, such epithelial–mesenchymal interactions are dysregulated due to epithelial regenerative failure or aberrant activation of fibroblasts. Myofibroblasts are the effector cells that are responsible for fibrogenesis and are characterized by the production of large quantities of ECM components, such as collagen and fibronectin, the secretion of proteases, and their contractile ability. Accumulating evidence suggests that YAP/TAZ signaling is linked to the pathophysiology of fibrosis, and aberrant YAP/TAZ activation has been reported in both the epithelial compartment and fibroblasts/myofibroblasts (Table 1).

### 4.1. Lung Fibrosis

Idiopathic pulmonary fibrosis (IPF) is a progressive chronic interstitial lung disease with a poor prognosis that is characterized by clusters of proliferating fibroblasts termed fibroblastic foci. Remodeling of the ECM and the contraction of fibroblasts contribute to tissue tension or stiffness, which is associated with decreased vital capacity in IPF patients. Liu et al. and our group showed that YAP/TAZ are highly expressed in the nucleus of lung fibroblasts in the fibroblastic foci of the lungs of patients with IPF, indicating that YAP/TAZ in lung fibroblasts play a significant role in lung fibrosis [34,35].

In cell culture experiments, YAP/TAZ accumulated in the nucleus of lung fibroblasts grown on a pathologically stiff matrix, but not on a physiologically compliant matrix. Fibroblasts on a stiff matrix showed increased proliferation, contraction, and ECM production compared to those on a soft matrix. These fibroblast responses to matrix stiffness were ablated by YAP/TAZ knockdown, suggesting that YAP/TAZ activation contributes to such myofibroblastic features. In a follow-up study, Liu et al. showed that the overexpression of the constitutively active mutant form of TAZ in lung fibroblasts on a soft matrix increased the expression of connective tissue growth factor (CTGF) and plasminogen activator inhibitor-1 (PAI-1, also known as SERPINE1), but not that of ECM proteins such as collagens and fibronectin. However, the expression of these ECM proteins was induced by TAZ overexpression in cells cultured on a stiff matrix. These findings indicate that the full activation of TAZ requires the input of mechanical stimuli to facilitate the expression of genes related to fibrosis [36]. Gene expression profiling by RNA-sequencing revealed that TAZ-regulated genes in lung fibroblasts were associated with cell migration and motility, and partly overlapped with those regulated by TGF-β, which is a known mediator of fibrogenesis [35].

**Figure 1:**
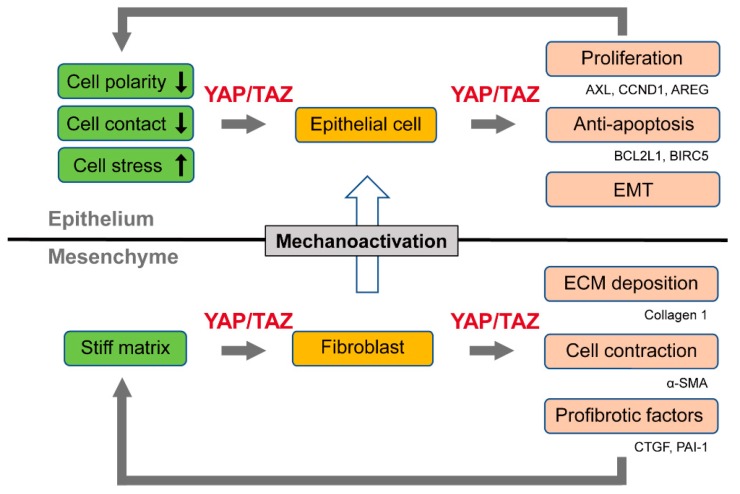
The activation of Yes-associated protein (YAP) and the transcriptional coactivator with PDZ-binding motif (TAZ) in epithelial cells and fibroblasts. In epithelial cells, the disruption of cell polarity, loss of cell contact, and increased cell stress signals activate YAP/TAZ, which promotes cell proliferation and the epithelial–mesenchymal transition (EMT), and inhibits apoptosis. In contrast, in fibroblasts, YAP/TAZ act as sensors of extracellular matrix (ECM) stiffness through the mechanotransduction pathway. YAP/TAZ also stimulate the production of fibrogenic factors and ECM proteins and enhance cell contraction. This process promotes tissue stiffness, thus forming a feed-forward loop of fibroblast activation and tissue fibrosis. YAP/TAZ can also be activated in epithelial cells of fibrotic tissues due to increased ECM stiffness.

Therefore, YAP/TAZ constitute a feed-forward loop that accelerates the fibrotic process. YAP/TAZ act as sensors of ECM stiffness, and their activity is enhanced by the progression of tissue fibrosis. In their roles as effectors, YAP/TAZ, in cooperation with TGF-β signaling, stimulate the production of fibrogenic factors (CTGF and PAI-1) and ECM proteins, thereby promoting the pathogenesis of tissue fibrosis (Figure 1).

Murine models of experimental pulmonary fibrosis support these profibrotic functions of YAP/TAZ. The heterozygous deletion of TAZ in mice led to resistance to lung fibrosis induced by the intratracheal administration of bleomycin [7]. Bleomycin-treated heterozygous mice had a lower tissue fibrosis score, hydroxyproline content, and lung elastance. Furthermore, the adoptive transfer of YAP/TAZ-overexpressing fibroblasts into the tail vein of wild-type mice led to elevated lung fibrogenic responses [34].

Regenerative failure of the respiratory epithelium following both acute and chronic injury is involved in lung fibrogenesis. YAP/TAZ signaling controls the proliferation and differentiation of epithelial progenitor cells in embryo and adult lungs [37]. Xu et al. performed single-cell RNA sequencing-based gene expression profiling of epithelial cells from IPF patients [38]. They identified distinct epithelial cell types with characteristics of conducting airway basal and goblet cells, and an additional subset of atypical transitional cells. Pathway analysis showed that the YAP/TAZ, TGF-β, Wnt, and PI3K signaling pathways were aberrantly activated. Immunofluorescence staining of the lung epithelial cells of IPF patients demonstrated increased nuclear YAP accumulation [39]. Interestingly, YAP was suggested to interact with mTOR/PI3K/AKT signaling to enhance the proliferation and migration, and inhibit the differentiation, of lung epithelial cells.

As such, YAP/TAZ activation in lung fibroblasts and epithelial cells differentially contributes to the pathogenesis of IPF. YAP/TAZ enhance fibrotic reactions while stimulating epithelial cell regeneration, albeit in a pathological manner (Figure 1).

### 4.2. Kidney Fibrosis

High TAZ expression was demonstrated in the kidneys of patients with IgA nephropathy or membranous nephropathy, both of which are pathologically characterized by renal tubulointerstitial fibrosis [40]. The YAP level is high in the renal tubular epithelium during the regeneration and fibrogenesis stages after acute kidney injury [41]. Increased TAZ nuclear accumulation in the renal tubulointerstitium was observed in three different mouse models of nephropathy (obstructive, diabetic, and toxin-induced renal injuries) [42]. Cell culture experiments involving renal fibroblasts showed that TGF-β-induced canonical Smad2/3 signaling is regulated by ECM stiffness in a manner dependent on YAP/TAZ [43].

In renal tubular epithelial cells, YAP overexpression promoted cell proliferation and YAP/TAZ activation induced by SAV1 deletion triggered epithelial–mesenchymal transition (EMT)-like phenotypic changes [40,41]. YAP/TAZ activation was also found in podocytes in a rat model of glomerular disease induced by intraperitoneal administration of puromycin aminonucleoside [44]. YAP overexpression in podocytes increased the levels of several ECM-related proteins—such as collagen 6, its receptor BCAM, and matrix metalloproteinase ADAMTS1—which promoted basement membrane thickening and stiffening.

### 4.3. Skin Fibrosis

YAP regulates keratinocyte proliferation and differentiation, and is essential for epidermal homeostasis and regeneration [45,46]. The nuclear accumulation of YAP/TAZ is reported in the dermis during the healing of skin wounds [47]. The closure rate of YAP/TAZ siRNA-treated skin wounds was significantly decreased in a mouse model, indicating that YAP/TAZ promote wound healing. YAP/TAZ modulated the expression of TGF-β signaling pathway components and its targets such as Smad2, p21, and Smad7.

YAP/TAZ are also activated in the dermal fibroblasts of patients with fibrotic diseases. Piersma et al. showed that YAP expression levels and nuclear localization were elevated in the skin tissue of patients with Dupuytren disease, which is a fibroproliferative disorder of the hands and fingers [48]. The knockdown of YAP in dermal fibroblasts attenuated the TGF-β-mediated formation of contractile actin stress fibers and the deposition of collagen type I. In addition, YAP/TAZ proteins were localized in the nucleus of fibroblasts in skin biopsies from patients with systemic sclerosis (SSc) [49]. Notably, dimethyl fumarate exerts a potent anti-fibrotic effect in SSc dermal fibroblasts by promoting the proteosomal degradation of YAP/TAZ.

### 4.4. Liver Fibrosis

Hepatic stellate cells (HSCs) are the dominant source of fibrogenic myofibroblasts in patients with chronic liver diseases. The nuclear localization of YAP was found in the stellate cells of mouse fibrotic liver induced by the administration of carbon tetrachloride and in human cirrhotic livers caused by infection with hepatitis C virus [50]. In vitro, siRNA-mediated silencing of YAP or the pharmacological inhibition of YAP with verteporfin, which interferes with the interaction between YAP/TAZ and TEAD, blocked the induction of the expression of its target genes and myofibroblast differentiation from HSCs. In vivo treatment with verteporfin reduced liver fibrogenesis in mice, further demonstrating that YAP is a key regulator of HSC activation. These findings were reinforced by a recent report that the hedgehog pathway-mediated activation of YAP directs HSC differentiation to myofibroblasts [51].

Wang et al. showed that TAZ activation in hepatocytes promotes progression from steatosis to non-alcoholic steatohepatitis (NASH) [52]. TAZ expression was increased in the hepatocytes of patients with NASH. TAZ silencing in hepatocytes prevented and even reversed hepatic inflammation and fibrosis in mouse models of NASH. Mechanistically, TAZ enhanced Indian hedgehog (Ihh) secretion from hepatocytes, which upregulated the expression of profibrotic genes, including collagen 1 and TIMP metallopeptidase inhibitor 1 (TIMP1), in HSCs.

## 5. YAP/TAZ Signaling in Cancer Cells

YAP/TAZ are upregulated and activated in a variety of human cancers. YAP/TAZ promote tumor initiation, progression, and metastasis. Furthermore, the higher expression or activation of YAP/TAZ is correlated with a poor prognosis, as reviewed in some articles [16,53,54,55,56,57,58]. Cell culture experiments showed that YAP/TAZ promote proliferation, anti-apoptosis, anchorage-independent growth, drug resistance, and stem cell traits in a variety of cancer cell lines. Xenograft mouse models and genetically engineered mice with tissue-specific overexpression or the deletion of YAP/TAZ or Hippo signaling components demonstrated a crucial role for YAP/TAZ in the formation and growth of tumors [53]. For example, hepatocyte-specific deletion of MST1/2 in mice resulted in YAP activation, leading to significantly enlarged livers and tumor development by the age of five to six months [59]. Available evidence concerning YAP/TAZ expression and functional relevance in cancer is outlined in Table 2.

Multiple mechanisms of YAP/TAZ activation have been reported in different cancer types. Several cancers harbor the amplification of YAP as part of the 11q22 amplicon [80]. TAZ-CAMTA1 or YAP-TFE3 gene fusions, which render the N-terminus of YAP/TAZ unresponsive to negative regulation by the Hippo pathway, have been reported in epithelioid hemangioendothelioma, which is a rare form of sarcoma [81,82]. A high frequency of inactivating mutation of NF2, which is a negative regulator of YAP/TAZ signaling, has been found in several types of tumor, such as meningioma, schwannoma, and malignant mesothelioma [83,84,85]. Except for NF2, DNA mutations in the Hippo pathway components are rare, and no activating mutation of YAP/TAZ has been demonstrated in human cancers. The epigenetic gene silencing of upstream negative regulators of YAP/TAZ such as MST1/2, LATS1/2, and RASSF1A has been reported in several cancers [86,87,88]. In most tumors, YAP/TAZ signaling can also be activated by a number of extrinsic signals that are enhanced in cancer tissues, which include increased mechanical forces, growth factors, inflammatory mediators, hypoxia, and altered metabolic conditions.

YAP/TAZ enhance tumor proliferation and survival by transactivating target genes associated with cell-cycle progression and anti-apoptosis. YAP physically interacts with mutant p53 protein and upregulates cyclin A (CCNA), cyclin B (CCNB), and CDK1 in cooperation with the NF-Y transcriptional factor in breast cancer cells [89]. YAP enhances the proliferation of malignant mesothelioma cells by directly upregulating cyclin D1 (CCND1) [90]. An EGFR ligand, amphiregulin (AREG), is also induced by YAP/TAZ, and stimulates cell proliferation and malignant transformation by activating EGFR signaling in a non-cell autonomous manner [60,62]. YAP and TBX5 form a complex with β-catenin and transactivate anti-apoptosis genes, including BCL2L1 and BIRC5, in various types of cancer cell [91]. Furthermore, YAP/TAZ promote cell cycle progression by inducing the expression of the proto-oncogene, c-Myc [92].

Recent genome-wide occupancy profiling studies revealed that YAP/TAZ binding sites are not restricted to gene promoters. Zanconato et al. found, by chromatin immunoprecipitation (ChIP)-sequence analysis of breast cancer cells, that most YAP/TAZ-bound cis-regulatory regions coincide with enhancer elements, which are located distant from transcription start sites [92]. Notably, the AP-1 transcription factor was present in most YAP/TAZ/TEAD-binding sites, forming a complex and synergistically activating target genes involved in cell cycle progression. Most recently, it has been shown that YAP/TAZ-bound enhancers recruit bromodomain-containing protein 4 (BRD4), which is a critical epigenetic modulator that binds to acetylated histone, boosting the expression of growth-regulating genes [93].

## 6. YAP/TAZ Signaling in the EMT

EMT involves the transformation of epithelial cells to mesenchymal cells. The EMT of cancer cells confers aggressive features such as invasion, resistance to apoptosis, and at advanced disease stages, chemoresistance [94]. During the EMT, epithelial cells lose their apical–basal polarity, basement membrane attachment, and cell–cell contact. In turn, they gain migratory and invasive properties associated with the mesenchymal phenotype [95]. The EMT is executed by a subset of transcription factors, including ZEB1/2, Snail/Slug, and Twist. In cancer cells, the EMT is also associated with cancer stem cell (CSC) characteristics, anti-apoptosis, and drug resistance [94]. The pathological significance of the EMT in tissue fibrosis is controversial, but the upregulation of EMT-related factors and the colocalization of epithelial and mesenchymal markers in fibrotic tissues have been demonstrated [96,97].

YAP/TAZ signaling is involved in the EMT. Lei et al. showed that the overexpression of TAZ in mammary epithelial cells induces the EMT [98]. YAP regulates multiple EMT-related genes by inducing SOX2 expression in cooperation with Oct4 in non-small lung cancer cells [99]. Moreover, YAP can rescue cell death induced by the suppression of KRAS in cancer cells [100,101]. YAP and FOS, a member of the AP-1 transcription factor family, coordinately regulate the EMT by directly binding to the promoter region of Vimentin and Slug.

Interestingly, the EMT itself can promote TAZ activation. Induction of the EMT by ectopic overexpression of Twist and Snail leads to the delocalization of Scribble, which is a scaffold protein involved in cell polarization, from the cell membrane. Next, TAZ is relieved from the Scribble polarity complex and promotes CSC traits in breast cancer cells [102]. These findings suggest that the TAZ-mediated EMT serves as a self-sustaining mechanism of TAZ activation. Increasing matrix stiffness can also induce the EMT and promote tumor invasion and metastasis in breast cancer cells, in which Twist plays an essential role as a mechanomediator [103].

ZEB1 directly binds to YAP to regulate the transcription of target genes [104]. In terms of clinical importance, the set of ZEB1/YAP target genes was a strong predictor of poor relapse-free survival, therapy resistance, and an increased risk of metastasis in breast cancer. Similarly, Snail/Slug form a complex with YAP/TAZ to regulate the self-renewal and differentiation of skeletal stem cells [105].

Therefore, YAP/TAZ in close association with ZEB1/2, Snail/Slug, and Twist govern the EMT, thereby inducing the malignant features of cancer cells. Indeed, YAP/TAZ confer CSC-related traits—such as tumor initiation, drug resistance, and metastasis—in a wide range of human cancers. The first evidence came from the work of Cordenonsi et al. [102]. This group showed that TAZ is highly expressed in the CD44^high^/CD24^low^ subpopulation with CSC properties in primary breast tumors. TAZ was required for self-renewal and the formation of high-grade tumors. The gene expression profiling of patient-derived breast cancer stem cell lines showed that TAZ is a central mediator of metastasis and resistance to chemotherapy [106]. YAP directly upregulates SOX9 and induces CSC properties in esophageal cancer cells [107]. YAP endows urothelial cancer cells with CSC traits by directly binding to the enhancer region of SOX2 [108]. Conversely, SOX2 directly represses two negative regulators of YAP/TAZ, NF2 and WWC1, leading to activation of YAP and maintenance of CSCs in osteosarcomas [109].

## 7. YAP/TAZ Signaling in the Cancer Microenvironment

The interactions between cancer cells and their supporting stroma are critical determinants of cancer initiation and progression. The structural components of tumor stroma, which constitute the tumor microenvironment, include ECM, blood and lymphatic vessels, and stromal cells including endothelial cells, immune cells, and cancer-associated fibroblasts (CAFs). Fibroblasts are activated by cancer-associated soluble factors such as TGF-β and produce ECM proteins. Collagens and other ECM proteins, including fibronectins, proteoglycans, and tenascin C, are overproduced in several cancers [110]. In addition, lysyl oxidase (LOX) and LOX-like (LOXLs) enzymes mediate the process of covalent intramolecular and intermolecular crosslinking of collagen fibers and other ECM components, resulting in increased tissue stiffness [111]. This desmoplastic stiff stroma is generally considered to enhance the proliferation and motility of cancer cells [112]. Indeed, ECM stiffness is correlated with the progression and a poor prognosis of cancer [111,113]. Moreover, treatment with the ECM-degrading enzyme hyaluronidase decreased interstitial pressure and reduced the aggression of pancreatic ductal cancer, suggesting that the ECM has potential as a therapeutic target for cancer [114].

As mentioned above, the activity of YAP/TAZ is regulated by ECM stiffness. The activation of YAP/TAZ by the stiff ECM of fibrotic tissues can promote the proliferation and survival of cancer cells. Jang et al. showed that YAP and TEAD directly control the mechanical cue-dependent transcription of Skp2, which is important for cell cycle progression in breast cancer cells [115]. Agrin, an ECM component, was reported to activate YAP by suppressing the Hippo pathway, leading to the development of liver cancer [116,117,118]. Agrin signals matrix and cellular rigidity by activating the integrin-FAK-ILK signaling axis. This, in turn, stimulates PAK1, which inactivates NF2, a negative regulator of YAP/TAZ. Agrin also activates YAP through RhoA-dependent actin cytoskeletal rearrangements. Agrin is secreted by platelet-derived growth factor (PDGF)-stimulated HSCs and promotes hepatocarcinogenesis [119]. Glypican-3, a member of the glypican family of heparan sulfate proteoglycans, is also involved in the progression of hepatocellular carcinoma (HCC). HN3, a conformation-specific antibody against glypican-3, inhibits the proliferation of HCC cells in vitro and in vivo by inducing cell-cycle arrest through YAP downregulation [120].

CAFs promote cancer invasion and metastasis by producing soluble factors and matrix remodeling [110]. A coculture experiment involving carcinoma cells and stromal fibroblasts revealed that CAFs promote the invasion of cancer cells by making passageways in the ECM [121]. CAFs generate a gap in the basement membrane and ECM by releasing matrix metalloproteinases and pulling ECM fibers via direct mechanical forces [122].

**Figure 2:**
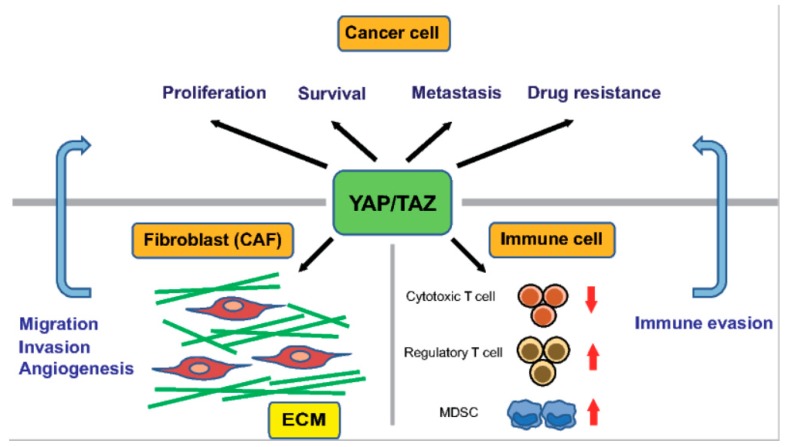
The function of YAP/TAZ in cancer cells and in the cancer microenvironment. YAP/TAZ enhance the proliferation, survival, metastasis, and drug resistance of cancer cells. The cancer microenvironment comprises ECM and stromal cells, such as cancer-associated fibroblasts (CAFs) and immune cells. The activation of YAP/TAZ in CAFs promotes migration and invasion of cancer cells, and angiogenesis. YAP/TAZ facilitate tumor immune evasion by suppressing cytotoxic T cells through PD-L1 expression in cancer cells, and supporting myeloid-derived suppressor cells (MDSCs) and regulatory T cells.

A stiff ECM enhances the activity of YAP/TAZ not only in cancer cells but also in stromal cells, including CAFs. Recently, Calvo et al. demonstrated that YAP/TAZ in CAFs play a crucial role in the progression of mammary tumors [123]. First, they performed a global mRNA expression profiling analysis of fibroblasts isolated from mice with breast cancer of various disease stages, and found that the gene signature of YAP/TAZ signaling is enriched in CAFs compared with in normal mammary fibroblasts (NFs). Consistently, YAP was predominantly cytoplasmic in NFs but active in the nucleus in CAFs. The siRNA-mediated silencing of YAP reduced the ability of CAFs to contract a collagen-rich matrix and promote invasion of cancer cells and angiogenesis. These findings indicate that YAP is required for the tumor-promoting functions of CAFs. Furthermore, a series of cell culture experiments revealed that the YAP-mediated activation of myosin light chain 2 is critical for generating CAFs, which subsequently promote matrix remodeling and tumor invasion. Moreover, a stiff ECM induced the formation of actin stress fibers in fibroblasts, leading to YAP activation, thus establishing a self-sustaining feed-forward loop to maintain the phenotype of CAFs (Figure 2).

The myocardin-related transcription factor (MRTF)—serum response factor (SRF) pathway is also involved in the response to mechanical stress and is under the control of Rho GTPase signaling. MRTF and SRF promote the expression of dozens of cytoskeleton-related genes, including that encoding α-SMA. CAFs exhibit elevated MRTF–SRF signaling, which is required for their contractile and pro-invasive properties [124]. The MRTF–SRF and YAP–TEAD pathways can indirectly activate each other by modulating actin cytoskeletal dynamics in CAFs.

Recent studies have uncovered new roles for YAP/TAZ signaling in the host immune response in the tumor microenvironment (Figure 2). Cancers escape from host immunity by upregulating PD-L1, the interaction of which with PD-1 receptors on activated T cells reduces the proliferative capacity and effector function of cytotoxic T cells. YAP/TAZ promote immune evasion by upregulating PD-L1 in cancer cells, via directly binding to its promoter with TEADs in human cancer cells [125,126]. Coculture experiments demonstrated that the overexpression of TAZ in cancer cells is sufficient to disrupt the functions of T cells by upregulating the expression of PD-L1. TAZ-dependent upregulation of PD-L1 can be induced by activation of lactate-mediated G protein-coupled receptor 81 (GPR81) [127].

YAP/TAZ also recruit myeloid-derived suppressor cells (MDSCs) by upregulating the production of proinflammatory cytokines, including CXCL5 and TNF-α [128,129]. The hepatocyte-specific deletion of MST1/2 in mice leads to increased macrophage infiltration and HCC formation in the liver. Recently, Kim et al. showed that monocyte chemoattractant protein 1 (MCP1) expression in hepatocytes are required for tumor formation in the MST1/2 double knockout mice [130]. MCP1 recruits macrophages with both M1 and M2 characteristics, which regulate hepatocyte proliferation and survival. MCP1 is a direct transcriptional target of YAP/TAZ in hepatocytes. Furthermore, in HCC, YAP promotes differentiation of naïve T cells to regulatory T cells by directly upregulating the expression of TGF-β receptor 2 (TGFBR2) [131]. Moreover, YAP is highly expressed in regulatory T cells and bolsters their suppression of anti-tumor immunity by activating Activin signaling [132].

## 8. Crosstalk with the TGF-β and Wnt Pathways

YAP/TAZ signaling cooperates with the TGF-β and Wnt pathways in epithelial cells and fibroblasts, which accelerates profibrotic reactions. Such crosstalk is presumed to be also active in cancer cells and CAFs, in which it promotes tumor progression.

### 8.1. Crosstalk with TGF-β Signaling

TGF-β signaling is closely associated with both tissue fibrosis and cancer [133]. TGF-β drives the differentiation of quiescent fibroblasts into matrix-secreting myofibroblasts, which is a key step in tissue fibrosis. As mentioned above, CAFs promote tumor progression by enhancing ECM deposition, angiogenesis, and secretion of growth factors. Importantly, TGF-β is a strong inducer of the CAF phenotype and TGF-β signaling is activated in CAFs [134,135]. Therefore, it is conceivable that fibroblast activation and ECM remodeling in tissue fibrosis promote tumor progression.

TGF-β suppresses the proliferation of epithelial cells and acts as a tumor suppressor during the early stages of tumorigenesis. However, TGF-β is also a potent inducer of the EMT. The activation of TGF-β signaling and EMT-related transcriptional changes are also observed in epithelial cells derived from IPF lung tissues [39]. It is tempting to speculate that epithelial cells undergoing the EMT in tissue fibrosis are primed to gain malignant features following carcinogenesis by mutations in oncogenic driver genes. TGF-β binds to TGF-β receptors type I and II, which then phosphorylate the intracellular signal transducers, Smad2 and Smad3. These form a heterotrimeric complex with Smad4 and translocate into the nucleus, resulting in activation of the expression of various genes. The interaction between TAZ and Smad2/3 is critical in nucleocytoplasmic shuttling of the Smad2/3-Smad4 complex [136]. In the absence of TAZ, the Smad complex fails to accumulate in the nucleus, and TGF-β-mediated transcription is disrupted in embryonic stem cells. RASSF1A regulates the TGF-β-induced interaction between YAP and Smad2, which suppresses the invasion of cancer cells [137].

The promoters of a variety of genes harbor both Smad-binding elements (SBEs) and TEAD-binding elements [138]. In the nucleus, YAP/TAZ, TEAD, and Smad2/3 form a complex that regulates the transcription of these genes. NEGR1 and UCA1 are synergistically activated by this complex, and are necessary for maintaining the tumorigenic activity of metastatic breast cancer cells. The YAP–TEAD–Smad complex also binds to the promoter of CTGF, which enhances ECM production and proliferation by malignant mesothelioma cells [139]. A recent study has shown that TGF-β increases the TAZ protein level in fibroblasts via Smad3-independent, and p38 and MRTF-mediated mechanisms [140].

### 8.2. Crosstalk with Wnt Signaling

Wnt signaling also plays a key role in fibrogenesis and carcinogenesis. The activation of Wnt signaling in epithelial cells and fibroblasts is a common feature of fibrotic tissues and contributes to the proliferative and migratory activities in various organs, including the lung, kidney, heart, and skin [3,141,142,143]. The inhibition of Wnt signaling by a small molecule, ICG-001, prevented and even reversed bleomycin-induced lung fibrosis in mice [144]. Aberrant Wnt signaling is also involved in carcinogenesis, as genetic alterations in Wnt signaling components are frequently observed in various cancers [145].

In the absence of Wnt ligands, β-catenin is captured by the cytoplasmic destruction complex, which consists of a scaffold protein, Axin, and other components such as APC, GSK3β, and CK1. This capture results in the phosphorylation and degradation of β-catenin by β-TrCP. In the presence of Wnt ligands, the destruction complex is dissociated, leading to the stabilization and nuclear accumulation of β-catenin. In the nucleus, β-catenin interacts with the transcription factor TCF/LEF to activate the transcription of target genes.

There is complex crosstalk between Wnt and YAP/TAZ signaling, and YAP/TAZ activity is indispensable for Wnt-induced biological responses [11,146]. Azzolin et al. showed that phosphorylated β-catenin serves as a presenting factor for TAZ to β-TrCP and promotes TAZ degradation [11]. Wnt signaling stabilizes β-catenin, which leads to nuclear accumulation of TAZ and enhancement of its transcriptional activity. Gene expression profiling revealed that the regulation of a considerable portion of Wnt target genes was TAZ-dependent in both mammary epithelial cells and colorectal cancer cells. In a follow-up study, they showed that YAP/TAZ are integral components of the β-catenin destruction complex [146]. The activity of YAP/TAZ is regulated by Wnt signaling in a similar manner to β-catenin and is required for stabilization of β-catenin.

Cai et al. reported that Wnt signaling mediates the β-catenin-independent activation of YAP/TAZ [147]. APC, a core component of the β-catenin destruction complex, interacts with SAV1 and LATS1, which are upstream regulators of YAP/TAZ. The regulation of YAP/TAZ signaling by APC is essential for intestinal tumorigenesis. DVL, a scaffolding protein involved in Wnt signaling, is required for the nucleocytoplasmic shuttling of YAP [148]. Wnt can also activate YAP/TAZ via the Frizzled (a GPCR-like Wnt receptor)–Gα12/13–Rho GTPases–LATS1/2 axis, which is independent of canonical Wnt/β-catenin signaling [149]. Moreover, YAP was suggested to be a direct transcriptional target of Wnt/β-catenin signaling, and its expression is required for the growth of colorectal cancer cells [150].

Multiple layers of crosstalk between Wnt signaling and YAP/TAZ activity have been reported, and these mechanisms may be involved in the pathogenesis of tissue fibrosis and the progression of cancers.

## 9. Conclusions

There is abundant epidemiologic evidence that fibrosis—including pulmonary fibrosis and liver cirrhosis—increases the risk of cancer in various organs. Among the mechanisms linking these two diseases, recent studies strongly suggest that YAP/TAZ signaling plays an important role. (1) YAP/TAZ contribute to fibrosis by acting both as mechanosensors and profibrotic effectors in fibroblasts/myofibroblasts. Fibroblast activation and fibrotic changes in tissue fibrosis are similar to cancer stromal reactions and provide a tumorigenic microenvironment. (2) Increased matrix stiffness in tissue fibrosis can mechanoactivate YAP/TAZ in epithelial cells, which promotes cell proliferation and survival. Other extrinsic signals, including oxidative stress and hypoxia, also activate YAP/TAZ and promote tumorigenesis. (3) YAP/TAZ signaling cooperates with the TGF-β and Wnt pathways in epithelial cells and fibroblasts, which may exert profibrotic and tumorigenic effects. (4) YAP/TAZ signaling, possibly in cooperation with TGF-β signaling, is capable of inducing the EMT. EMT-related alterations in epithelial cells in fibrotic tissue may promote tumorigenesis. In cancer cells, the YAP/TAZ-mediated EMT may further promote tumorigenicity and cancer stemness.

YAP/TAZ signaling has potential as a target for anti-fibrosis and anti-cancer therapies. For example, by high-throughput screening, verteporfin was identified as a small molecule inhibitor of the interaction between YAP/TAZ and TEAD, and reversed the malignant behavior of cancer cells, although many off-target effects and general toxicity diminish its therapeutic potential [151,152]. A cell-based screen of chemical reagents that induce the recruitment of YAP to the cytosol showed that dobutamine inhibits the YAP-dependent gene transcription [153]. Rho and ROCK inhibitors abolish YAP/TAZ nuclear localization and activation [24]. Several studies have suggested that both fibrosis and cancer could be treated by inhibiting Rho and ROCK [154,155]. Statins, which downregulate YAP/TAZ activity via the mevalonate metabolic pathway, delay fibrosis progression and reduce the risk of liver cancer in patients with HCV infection [156]. As YAP/TAZ directly upregulate PD-L1 in cancer cells, PD-1/PD-L1 checkpoint inhibitors, standard therapies for various types of cancer, could be effective against cancers with YAP/TAZ activation.

However, targeting YAP/TAZ signaling may cause adverse effects because YAP/TAZ play critical roles in organ homeostasis and regeneration. Therefore, molecular partners directing their tumorigenic functions, such as AP-1, can also be promising therapeutic targets. Further studies are needed to identify the group of patients with tissue fibrosis or cancer that is most likely to benefit from therapies targeting YAP/TAZ signaling.

## Figures and Tables

**Table 1:** YAP/TAZ expression and functional relevance in organ fibrosis.

Organ	Expression in Human Tissues	Animal Model	Cell Culture (Cell Type)	Phenotype	Related Molecule	Reference
Lung	Elevated YAP/TAZ nuclear staining in lung fibroblasts of IPF patients Elevated YAP nuclear staining in lung epithelial cells of IPF patients	Intratracheal administration of bleomycin Adoptive transfer of fibroblasts into the tail vein	Lung fibroblast	Cell proliferation, contraction, ECM production, migration, myofibroblast differentiation	CTGF, PAI-1, Collagen 1, α-SMA	[7,34,35,36,39]
			Lung epithelial cell	Cell proliferation, migration, loss of apical–basal polarity	AXL, CTGF, AJUBA, Scribble, VANGL1	
Kidney	Elevated TAZ nuclear staining in the renal tubular epithelium and interstitium of patients with IgA nephropathy and membranous nephropathy Elevated YAP nuclear staining in the renal tubular epithelium during the regeneration and fibrogenesis stages after acute kidney injury (AKI)	Unilateral ureteral obstruction Streptozotocin-induced renal injury (diabetic nephropathy) Aristolochic acid-induced nephropathy Ischemia/reperfusion by clamping the bilateral renal arteries (AKI–chronic kidney disease transition)	Renal fibroblast	ECM production, myofibroblast differentiation	α-SMA, PAI-1, Collagen 1	[40,41,42,43,44]
			Renal tubular epithelial cell	Cell proliferation, EMT-like phenotype, ECM production	TGF-β2, TGF-β receptor 2, CTGF	
			Podocyte	ECM production	Collagen 6, BCAM, ADAMTS1	
Skin	Elevated YAP nuclear staining in the skin nodules of patients with Dupuytren disease Elevated YAP/TAZ nuclear staining in dermal fibroblasts of patients with SSc	Wound generation by full-thickness punch biopsy (wound healing process) Administration of bleomycin by osmotic minipump	Dermal fibroblast	Contraction, myofibroblast differentiation, ECM production	α-SMA, Collagen 1	[46,47,48,49]
			Keratinocyte	Cell proliferation, anti-apoptosis, suppressed differentiation	CYR61	
Liver	Elevated YAP nuclear staining in the stellate cells of human cirrhotic livers caused by infection with hepatitis C virus	Intraperitoneal administration of carbon tetrachloride Non-alcoholic steatohepatitis (NASH) model induced by diet rich in fructose, palmitate, and cholesterol	Hepatic stellate cell	Myofibroblast differentiation, cell proliferation	α-SMA, Collagen 1, CTGF, Glutaminase, MMP2	[50,51,52]
			Hepatocyte	Secretion of profibrotic factors	Indian hedgehog	

**Table 2:** YAP/TAZ expression and functional relevance in cancer.

Organ	Expression in Human Tissues	Animal Model	Cell Culture (Phenotype)	Related Molecule	Reference
Lung	Elevated YAP/TAZ expression correlates with advanced TNM stage and lymph node metastases and is a predictor of worse prognosis in non-small cell lung cancer.	TAZ knockdown impairs the growth of subcutaneous xenografts of the lung adenocarcinoma cell line A549 in mice. YAP deletion inhibits the progression of lung adenocarcinoma in LKB1-deficient Kras^G12D^ mice.	Cell proliferation, anchorage-independent growth, resistance to EGFR-tyrosine kinase inhibitor (EGFR-TKI)	AXL, CYR61, AREG, EREG, NRG1	[60,61,62,63,64,65]
Kidney	YAP expression is elevated in clear cell renal cell carcinoma (ccRCC) and mucinous tubular and spindle cell carcinoma. Elevated YAP expression is a predictor of worse prognosis in ccRCC.	YAP knockdown impairs the growth of subcutaneous xenografts of the renal cell adenocarcinoma cell line ACHN in mice.	Cell proliferation, migration, anchorage-independent growth	CYR61, c-Myc, Endothelin 1/2	[66,67,68,69,70]
Skin	Elevated YAP/TAZ expression correlates with tumor thickness and lymph node metastases and is a predictor of worse prognosis in melanoma.	YAP/TAZ knockdown inhibits lung metastasis following tail-vein injection of the melanoma cell line 1205Lu in mice. Pharmacological inhibition of YAP/TAZ with verteporfin impairs the growth of subcutaneous xenografts of the BRAF inhibitor-resistant melanoma cell line in mice.	Cell proliferation, invasion, anchorage-independent growth, resistance to BRAF and MEK inhibitors, immune evasion	CTGF, AXL, PD-L1	[71,72,73,74]
Liver	Elevated YAP/TAZ expression correlates with advanced TNM stage and poor tumor differentiation and is a predictor of worse prognosis in hepatocellular carcinoma (HCC). YAP expression is elevated in cholangiocarcinoma and hepatoblastoma. Elevated YAP expression correlates with poor response to transarterial chemoembolization in HCC.	Liver-specific YAP overexpression leads to hepatomegaly followed by liver tumor formation in mice. YAP/TAZ knockdown impairs the growth of subcutaneous xenografts of the hepatocellular carcinoma cell line Bel7402 in mice.	Cell proliferation, migration, invasion, EMT, resistance to irinotecan	BIRC5, c-Myc, ABCB1, ABCC1, FOXM1, Jag-1	[75,76,77,78,79]
